# Ferrostatin-1 alleviates cytotoxicity of cobalt nanoparticles by inhibiting ferroptosis

**DOI:** 10.1080/21655979.2022.2042143

**Published:** 2022-02-24

**Authors:** Weinan Zhang, Chen Wang, Wenfeng Zhu, Fan Liu, Yake Liu

**Affiliations:** a Department of Orthopaedics, Affiliated Hospital of Nantong University, Nantong, Jiangsu Province, China; b Department of Orthopaedics, The Sixth Affiliated Hospital of Nantong University, Yancheng, Jiangsu Province, China

**Keywords:** Cobalt nanoparticles, ferroptosis, Ferrostatin-1, hip arthroplasty, lipid peroxidation

## Abstract

Cobalt is the main component of metal prostheses in hip arthroplasty. Studies have shown that metal particles mainly composed of cobalt nanoparticles (CoNPs) can cause systemic and local toxic reactions due to various physical and chemical factors. Therefore, elucidating the underlying mechanisms of metal prosthesis action, coupled with identification of effective detoxification drugs are imperative to minimizing postoperative complications and prolonging the service life of these clinical tools. In this study, we treated Balb/3T3 mouse fibroblast cell line with CoNPs and ferrostatin-1, then measured cell viability via the CCK-8 assay. Next, we determined levels of reactive oxygen species (ROS), malondialdehyde (MDA), glutathione (GSH), cobalt and iron contents, as well as glutathione peroxidase 4 (GPX4), and solute carrier family 7 member 11 (SLC7A11) expression in each group. Finally, we employed transmission electron microscopy (TEM) to detect changes in the ultrastructure of each group of cells. Exposure of cells to CoNPs significantly suppressed their viability, and downregulated expression of GSH, GPX4, and SLC7A11 proteins. Conversely, this treatment mediated a significant increase in ROS, MDA, cobalt, and iron levels in the cells. TEM images revealed a marked increase in density of the mitochondrial membrane of cells in the CoNPs group, while the outer membrane was broken. Notably, treatment with ferroptosis inhibitor Ferrostatin-1 alleviated the cytotoxic response caused by CoNPs. These findings suggest that CoNP-induced cytotoxicity may be closely related to ferroptosis, indicating that inhibition of ferroptosis is a potential therapeutic strategy for reducing CoNP toxicity.

## Graphical abstract

**Figure:**
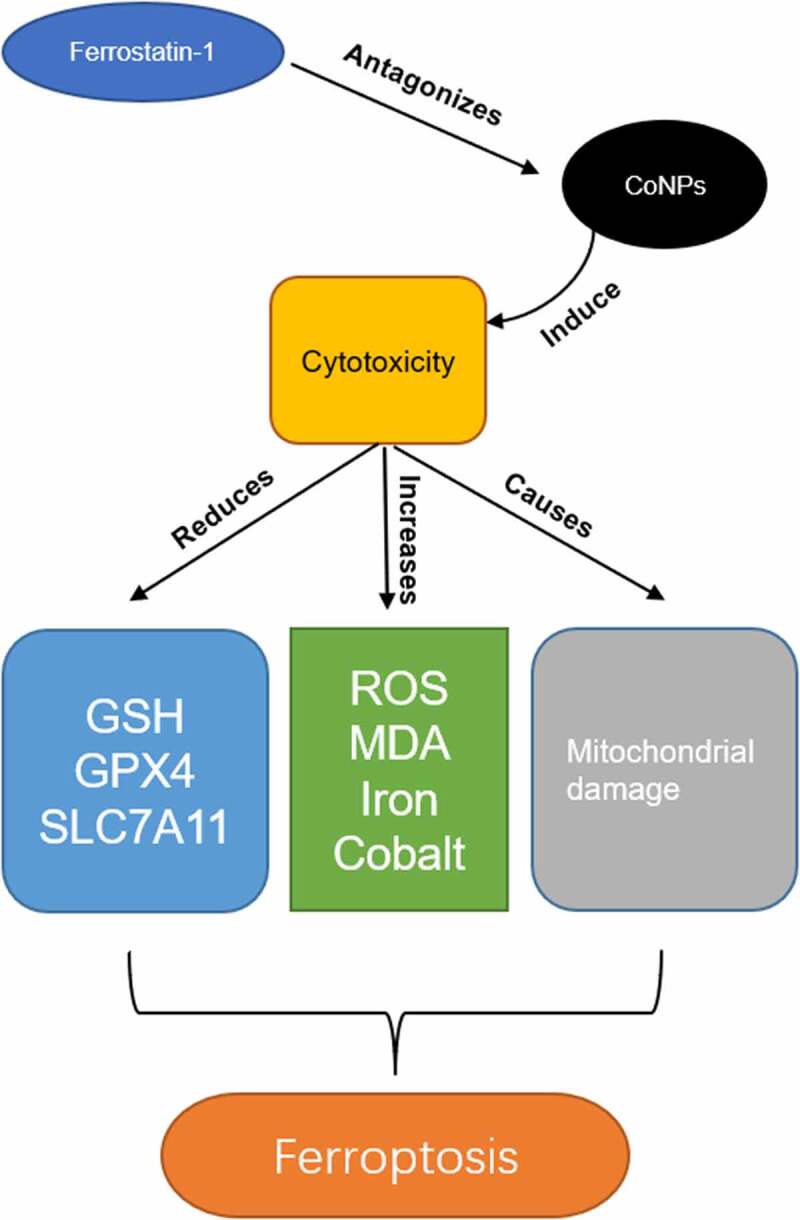

## Introduction

Millions of people around the world suffer from hip arthritis, with total hip replacement shown to be an effective clinical therapy for treating patients with end-stage arthritis. Since the mid-1980s, more than 1 million hip replacement prostheses, made of cobalt-chromium (CoCr) alloys, have been implanted worldwide [1]. Long-term implantation of metal endophytes causes Co^2+^ and Cr^3+^ ions to be released by metal particles in the bearings of hip implants, due to influence by physical and chemical factors, such as pH changes, fatigue, wear, fretting, and mechanical stress, among others. This release is caused by crevice corrosion of mechanically assisted tapered connection modules, including head-neck and neck-shank tapered interfaces [2,3]. Previous studies have proved that wear and corrosion particles extracted from surrounding tissues of devices are mainly in the nanometer size range. Notably, the particles are generally smaller than 50 nm, with a round and irregular morphology [4].

Patients undergoing total hip replacement manifest femoral osteolysis, with 29% of these patients exhibiting elevated levels of Co^2+^ in the blood during long-term follow-up [5]. This is because CoNPs are easily taken up by macrophages in tissues and transported to the phagolysosomes in the cells, which predisposes them to lysis and a large amount of Co^2+^ [6]. Previous studies have shown that Co^2+^ can not only inhibit expression of enzymes and proteins involved in DNA maintenance and repair, but also induce generation of ROS through a Fenton-like reaction in the presence of hydrogen peroxide to cause cell death [7]. Consequently, this causes a series of symptoms of poisoning in the nervous system, cardiovascular system, endocrine system, etc [2^,8–10^]. Therefore, oxidative stress is an important prerequisite for CoNP-induced cytotoxicity.

The term ferroptosis, coined in 2012, describes an iron-dependent, non-apoptotic form of cell death induced by Erastin and RSL3 [10]. Accompanying this discovery was identification of the first small-molecule ferroptosis inhibitor called Ferrostatin-1, an aromatic amine that specifically combines with lipid ROS to protect cells from lipid peroxidation-induced damage [11]. The mechanism underlying ferroptosis action can be roughly divided into two parts, namely the core pathway of ferroptosis and iron metabolism. Specifically, the core pathway that causes ferroptosis entails accumulation of lipid ROS caused by the inhibition of the cystine/glutamate antiporter system (system Xc^–^) and GPX4 [12]. Although iron is an essential component for normal functioning of cells, excessive accumulation of free Fe^2+^ can induce ROS production via the Fenton reaction, thereby causing excessive accumulation of fatal lipid peroxides that cause oxidative damage to cells [13,14]. Previous studies have shown that cells exposed to CoNPs exhibit a significantly lower total GSH content during the early stage, and a marked lipid peroxidation effect [15]. More recently, A research has shown that CoNPs can trigger ferroptosis-like cell death in neuronal cells [16]. Notwithstanding the studies conducted to date fall short in terms of providing a detailed understanding of the mechanism of cytotoxicity of CoNPs.

Herein, the present study was aimed to investigate Whether Ferrostatin-1 can alleviate cytotoxicity of CoNPs via inhibiting ferroptosis. We hypothesized that ferroptosis plays a key role in CoNP-induced cytotoxicity, while the ferroptosis inhibitor Ferrostatin-1 can effectively suppress this cytotoxicity.

## Materials and methods

### Characterization and preparation of CoNPs

Characterization of CoNPs (25–30 nm, Alfa Aesar, China) was as described in our previous study. Briefly, the diameter of CoNPs as measured by transmission electron microscope (TEM, JEM-2100 F, Japan), high-resolution scanning electron microscope (Hitachi 550 ultra-high-resolution SEM), and X-ray diffraction (XRD) was about 30 nm [17]. CoNPs were weighed, a day before the experiment, surface sterilized for 4 h at 180°C, and suspended in ultrapure water to prepare a stock solution with a concentration of 40 mM. The solution was sonicated, for 30 min, with an ultrasonic oscillator to disperse the CoNPs, diluted to the target concentration using complete culture medium, then quickly added to the cell sample. Ferrostatin-1 (#HY-100579, MCE) was dissolved in DMSO, at a concentration of 1 mM.

### Cell line and cell culture

The mouse fibroblast cell line, Balb/3T3, was purchased from the Chinese Academy of Sciences (Shanghai, China) and cultured in DMEM medium supplemented with 10% FBS, and 1% penicillin and streptomycin. All cells were grown in a humidified cell culture incubator, maintained at 5% CO_2_ and a temperature of 37°C. The medium was changed every day, and passaged every 2 to 3 days [18].

### Analysis of cell viability

Cell viability was determined using the CCK-8 assay kit (#CK04, Dojindo) according to the manufacturer’s instructions. Briefly, cells were seeded into a 96-well plate, then 10 μl of the CCK-8 reagent was added to each well and incubated for 2 h in cell culture incubator. Absorbance was then measured at 450 nm, the IC_50_ (the half maximal inhibitory concentration) value of CoNPs calculated, and the optimal detoxification concentration of Ferrostatin-1 determined [19].

### Detection of intracellular cobalt concentration

The Balb/3T3 cells were seeded into a 6-well plate (2 × 10^5^ cells/well) for 24 h, then treated with CoNPs (400 μM) and Ferrostatin-1 (1 μM) for 24 h. Next, the cells washed 3 times with PBS, digested with trypsin via centrifugation and the cell pellet collected. The cells were resuspended in 1 ml PBS, then counted using a cell counter (Thermo Fisher, USA). The cells were centrifuged, supernatant discarded, then lysed on ice for 20 min with 500 μl of lysis buffer. Next, the contents were centrifuged for 20 min (4°C, 12,000 rpm/min), the supernatant collected, and Cobalt concentration in supernatants of samples in each group determined via Inductively coupled plasma mass spectrometry (ICPMS, PerkinElmer NexION 350).

### Evaluation of intracellular iron content

Balb/3T3 cells were seeded into a 6-well plate (2 × 10^5^ cells/well) for 24 h, treated with CoNPs (400 μM) and Ferrostatin-1 (1 μM) for 24 h. Next, cells were washed 3 times with PBS, digested with trypsin and the collected. The cells were resuspended in 1 ml PBS, and their number counted using a cell counter. Next, the cells were centrifuged, the supernatant discarded, and the pellet lyzed with 500 μl lysis buffer on a shaker for 2 h. Finally, intracellular Fe^2+^ and total iron concentrations were determined using the Iron Assay Kit (#ab83366, Abcam), according to the manufacturer’s instructions.

### Measurement of Reactive Oxygen Species

Balb/3T3 cells were seeded into a 6-well plate (2 × 10^5^ cells/well) for 24 h, treated with CoNPs (400 μM) and Ferrostatin-1 (1 μM) for 24 h, then incubated with the DCFH-DA probe in the ROS kit (#S0033S, Beyotime) for 30 min in the dark. Next, the cells were washed 3 times with PBS, and observed under an inverted fluorescence microscope (Olympus, Japan) at excitation (Ex) and emission (Em) wavelengths of 485 nm and 525 nm, respectively.

### Evaluation of malondialdehyde and glutathione

Balb/3T3 cells were seeded and treated mentioned above, collected, and counted on a cell counter. Next, c cellular concentrations of MDA and GSH were measured using MDA and GSH kits (#BC0020; #BC1175, Solarbio) according to the manufacturer’s instructions [20].

### Detection of mitochondrial membrane potential

The Balb/3T3 cells were seeded and treated as described above. Cell culture medium was aspirated out, and the cells incubated with Mito-Tracker Red CMXRos and Hoechst 33,342 in the Mito-Tracker Red CMXRos staining solutions, as described by the manufacturer’s instructions of the Mitochondrial Membrane Potential Kit (# C1071S, Beyotime) at room temperature in the dark for 20–30 min. The contents were immediately place in an ice bath, then subjected to inverted fluorescence microscopy for detection of cell fluorescence. Mito-Tracker Red CMXRos and Hoechst 33,342 were detected as red and blue fluorescence, respectively [21].

### Western blot analysis

Balb/3T3 cells were first washed twice with PBS, then total proteins extracted from them using the RIPA reagent containing 1% PMSF. Protein concentration was determined using a Nanodrop one (Thermo Fisher, USA). Equal concentrations of proteins across samples were separated on a 12% SDS-PAGE gel electrophoresis, and transferred to polyvinylidene difluoride (PVDF) membranes (#FFP24, Beyotime). The membranes were blocked, at room temperature, with 5% skimmed milk powder diluted in TBST for 2 h, then incubated overnight with the following primary antibodies at 4°C: anti-GPX4 antibody (1:1000, #ab125066, Abcam), Anti-xCT antibody (1:1000, #ab37185, Abcam), and anti-GAPDH antibody (1:1000, #ab59164, Abcam). The membranes were washed with TBST, then incubated with the Anti-Mouse IgG secondary antibody (1:5000, #L3202, SAB) for 2 h at room temperature. Finally, the blots were stained with a chemiluminescence reagent (#P10300, NCM) and protein expression quantified using the ImageJ software [22].

### Transmission electron microscopy

Distribution of CoNPs, inside and outside the cell, as well as changes in cell ultrastructure were detected via TEM. Briefly, cells were treated with CoNPs (400 μM) and Ferrostatin-1 (1 μM) for 24 h, washed twice with PBS, collected via centrifugation and added to the agar solution. After solidification of the agar, cell clumps were fixed with 2.5% glutaraldehyde in 0.1% sodium chloride buffer, rinsed, dehydrated, embedded, and sectioned [23]. Finally, changes in cell ultrastructure were detected under a transmission electron microscope.

### Statistical analysis

Data, from at least 3 replicates, were statistically analyzed using GraphPad Prism 9 software and expressed as means with their respective standard deviations (SD). Differences among groups were determined using a one-way analysis of variance (ANOVA), accompanied by a Dunnett’s test. Data followed by P < 0.05 were considered statistically significant.

## Results

### *CoNP cytotoxicity and ferrostatin-1ʹs detoxification effect* in vitro

**Figure 1.**
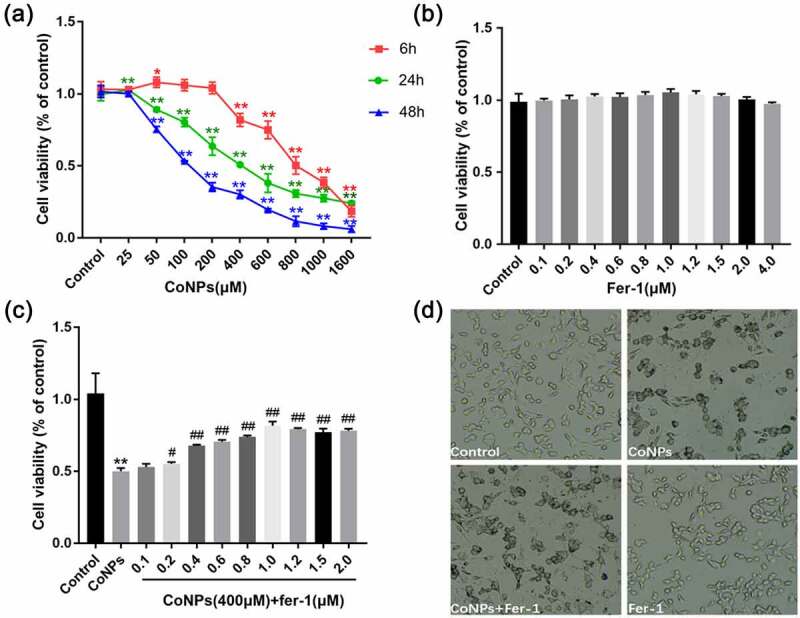
Ferrostatin-1 alleviates CoNP-induced cytotoxicity in Balb/3T3 cells. (a) Viability of cells incubated with different CoNPs concentrations after 6, 24, and 48 hours. (b) Cell viability after 24 h of treatment with different concentrations of Ferrostatin-1. (c) Viability of cells incubated with different concentrations of Ferrostatin-1 and CoNPs (400 μM) after 24 h of treatment. (d) Number and state of cells after 24 h of treatment with Ferrostatin-1 (1 μM)and CoNPs (400 μM). All the data are shown as mean ± SD of at least 3 replicates. *p < 0.05, **p < 0.01 versus the control group. ^#^p < 0.05, ^##^p < 0.01 versus the CoNPs group.

Results of the CCK-8 assay, after treatment of Balb/3T3 cells with different concentrations of CoNPs, revealed that low CoNPs concentrations had no significant effect on cell viability after 6 h of incubation, and cell viability only decreased significantly after increasing the concentration to more than 800 μM. In contrast, cell viability began to significantly decrease after 48 h of incubating the cells with 200 μM CoNPs. Notably, we observed a steady decrease in cellular activity with increase in CoNP concentration after 24 h (Figure 1a). Therefore, we based on cell viability at 24 h and calculated the IC_50_ value of CoNPs to be about 400 μM, then used CoNP concentration of 400 μM in subsequent experiments. Exposure of Balb/3T3 cells with different concentrations of Ferrostatin-1 for 24 h resulted in no significant change in cell viability, compared with the control group (Figure 1b). Subsequently, we used 400 μM CoNPs and different concentrations of Ferrostatin-1 to treat the cells over a 24-h period, and found that the cell survival rate was highest at 1 μM Ferrostatin-1 (Figure 1c-d). This was chosen as the optimal detoxification concentration of Ferrostatin-1.

### Ferrostatin-1 reduces levels of ROS and MDA in CoNP-treated Balb/3T3 cells

**Figure 2.**
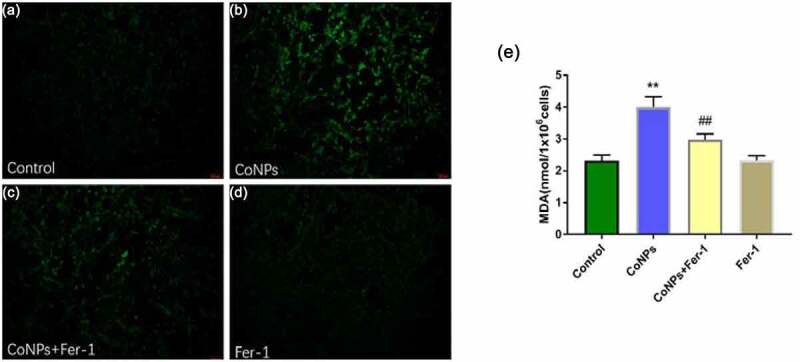
Ferrostatin-1 suppresses CoNP-induced production of ROS and MDA in Balb/3T3 cells. Cells were treated with CoNPs (400 μM) and Ferrostatin-1 (1 μM) for 24 h. (a-d) Observation of cells under an inverted fluorescence microscope (magnification = 400X), and the green fluorescence was the ROS detected by the DCFH-DA probe. (e). Intracellular MDA content in different treatment groups. All the data are shown as mean ± SD of at least 3 replicates. **p < 0.01 versus the control group. ^##^p < 0.01 versus the CoNPs group.

Ferroptosis mainly manifests as lipid peroxidation caused by accumulation of ROS. Therefore, we quantified levels of intracellular ROS and concentration of lipid peroxidation product MDA. Results showed that Balb/3T3 cells treated with CoNPs (400 μM) for 24 h significantly elevated ROS and MDA levels relative to the control group, while addition of Ferrostatin-1 effectively reversed this phenomenon (Figure 2a-e).

### Ferrostatin-1 upregulates GSH, GPX4, and SLC7A11 expression in CoNP-treated Balb/3T3 cells

**Figure 3.**
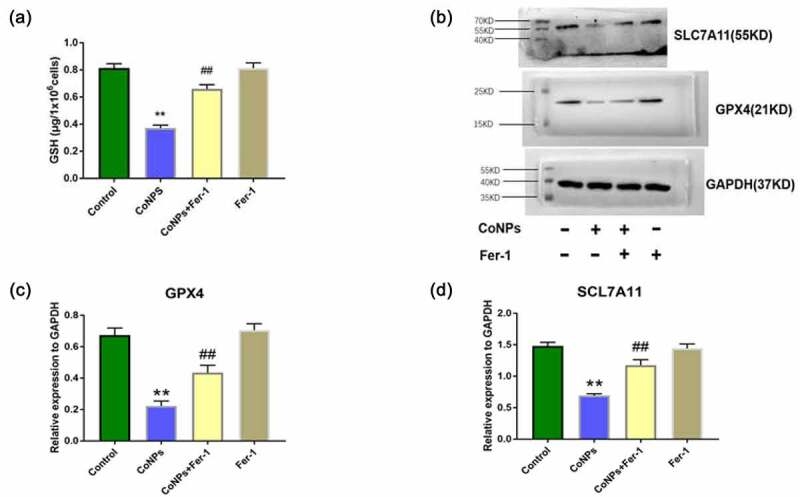
Ferrostatin-1 improves CoNPs-induced expression of GSH, GPX4, and SLC7A11 proteins in Balb/3T3 cells. (a) Levels of intracellular GSH across different treatment groups. (b-d) Western blots showing expression of GPX4 and SlC7A11 proteins. All the data are shown as mean ± SD of at least 3 replicates. **p < 0.01 versus the control group. ^##^p < 0.01 versus the CoNPs group.

Previous studies have shown that system Xc ^–^ enters cystine, which is reduced to cysteine during GSH synthesis, while GPX4 uses GSH to eliminate lipid peroxides formed by phospholipids containing polyunsaturated fatty acids [24]. In the present study, we detected intracellular GSH via spectrophotometry, and found that the intracellular GSH was significantly reduced in the CoNPs group alone, compared to the control group, while addition of Ferrostatin-1 could effectively inhibit GSH depletion (Figure 3a). In addition, Western blots showed that CoNP treatment significantly downregulated expression of GPX4 and SLC7A11 proteins, after 24 h relative to the control group. Conversely, addition of Ferrostatin-1 reversed this phenomenon (Figure 3b-d).

### Effect of Ferrostatin-1 on cobalt and iron content in CoNP-treated Balb/3T3 cells

**Figure 4.**
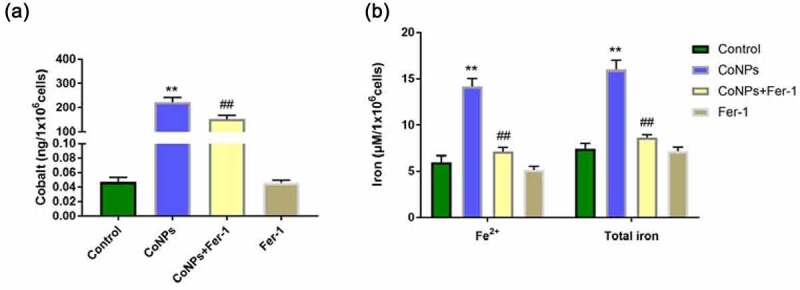
Ferrostatin-1 treatment significantly affects Cobalt and Iron concentration in Balb/3T3 cells. (a) ICP-MS spectrum showing the concentration of Cobalt in cells across different treatment groups. (b) Iron concentrations in cells across different treatment groups after iron assay. All the data are shown as mean ± SD of at least 3 replicates. **p < 0.01 versus the control group. ^##^p < 0.01 versus the CoNPs group.

CoNPs’ affinity to bind to the plasma membrane, coupled by its uptake by cells, are necessary prerequisites for development of CoNP toxicity [25]. Here, we measured levels of cobalt in cells using ICP-MS and found that this content significantly increased after 24 h of CoNPs treatment relative to the control group. However, addition of Ferrostatin-1 reduces cellular cobalt uptake ability in cells (Figure 4a). Iron overload has been implicated in lipid peroxidation and development of ferroptosis. We employed an Iron Assay Kit to determine iron content in cells and found that those in the CoNPs treatment group recorded significantly higher intracellular Fe^2+^ and total iron concentrations than the control group. However, cells exposed to both CoNPs and Ferrostatin-1 recorded significantly lower concentrations of Fe^2+^ and total iron than those treated with CoNPs alone (Figure 4b).

### Ferrostatin-1 suppresses CoNP-induced mitochondrial damage in Balb/3T3 cells

**Figure 5.**
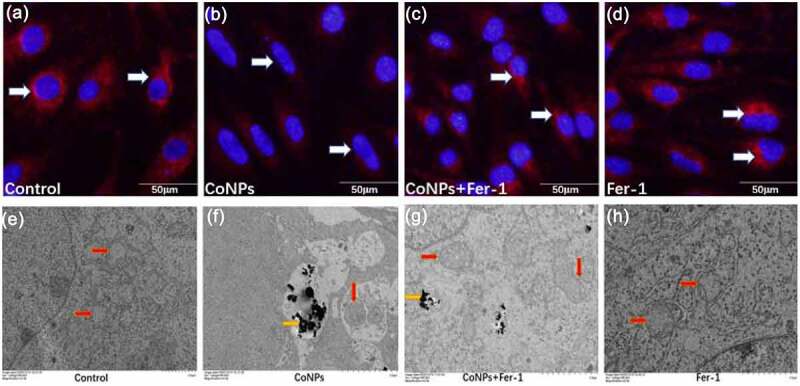
Ferrostatin-1 protects Balb/3T3 cells from CoNPs-induced toxic damage. (a-d) Cell observation under an inverted fluorescence microscope (magnification = 400X). Red fluorescence denotes mitochondrial membrane potential detected by the MitoTracker Red probe. (e-h) Ultrastructure of cells as observed under a TEM. The yellow arrows indicate CoNPs in the cytoplasm, while the red arrows indicate the mitochondria.

Previous studies have shown that ferroptosis can cause damage to the cell’s mitochondria, thereby causing a decrease in mitochondrial membrane potential [26]. Results of the present study showed that treatment of cells with CoNPs significantly lowered their mitochondrial membrane potential o compared to those in the control group. However, addition of Ferrostatin-1 significantly inhibited the decrease in mitochondrial membrane potential caused by CoNPs (Figure 5a-d). Ferroptosis in cells under electron microscopy is mainly manifested as mitochondrial damage, while there are almost no other obvious changes in cellular morphology prior to its death [10,27]. Results of the present study revealed that cells in the control and the Ferrostatin-1 groups had normal mitochondrial structure. Compared to the control group, treatment with CoNPs alone caused the mitochondrial outer membrane of the cells to break, and this was accompanied by an increase in membrane density, as well as disappearance of mitochondrial cristae. Results from the co-treatment group of CoNPs and Ferrostatin-1 showed that although cells still had some visible increase in mitochondrial membrane density, there was no membrane fragmentation (Figure 5e-h). This indicates that Ferrostatin-1 can protect cells from CoNP-induced cytotoxic damage to the ultrastructure.

## Discussion

Hip replacement is usually accompanied by deposition of metal wear particles in the surrounding tissues of the prosthesis caused a series of adverse reactions [28]. Although clinical therapies for treatment of acute cobalt poisoning have mainly focused on plasma exchange, or use of multiple chelating agents to reduce cobalt concentration in blood, the therapeutic effect remains unclear. Adverse reactions caused by chronic cobalt poisoning are mainly controlled through repair and removal of prostheses. However, surgery is not an efficacious treatment option for elderly patients or those that cannot withstand revision surgery [29,30]. Therefore, elucidating the underlying mechanism of CoNP-induced cytotoxicity and identification of efficacious detoxification methods are imperative to effective management of the condition. Results from our previous studies have confirmed that CoNPs can induce peroxidation through excessive production of ROS, which leads to apoptosis of Balb/3T3 cells, while the associated cytotoxicity can be inhibited using antioxidants [31]. However, in-depth research revealed that apoptosis is not the main mechanism of CoNP-induced cell death in Balb/3T3. We detected ferroptosis indicators in Balb/3T3 cells treated with Alpha lipoic acid and CoNPs, and found that CoNPs disrupted iron stability and caused the accumulation of lipid peroxides [32]. Therefore, apart from apoptosis, CoNP-induced cytotoxicity can also cause ferroptosis.

Ferroptosis, a form of cell death characterized by accumulation of lipid ROS and iron in cells, has been detected in multiple system diseases, such as cancer, subarachnoid hemorrhage, lung and kidney injury, and osteoarthritis [^33–38^]. Ferrostatin-1, a highly effective free radical trapping antioxidant (RTA) compound, has been shown to effectively inhibit ferroptosis and is associated with excellent protective efficacy across both *in vivo* and *in vitro* disease models [^39–41^]. To date, nothing is known regarding the mechanism through which CoNPs cause ferroptosis. To our knowledge, this is the first report describing Ferrostatin-1 as an antidote to CoNP-induced toxicity.

Summarily, our experimental results showed that low CoNPs concentrations can stimulate growth of Balb/3T3 cells, but an increase in concentration induces marked toxic effect on cells. Conversely, addition of Ferrostatin-1 significantly alleviates these toxic effects. ROS accumulation causes an increase in lipid peroxidation products, which is an important feature of ferroptosis. Results from the present study showed that Ferrostatin-1 can significantly reduce CoNP-induced ROS levels in Balb/3T3 cells as well as MDA production. Previous studies have shown that system Xc ^–^ is an important part of the cell synthesis of GSH, while GPX4 uses GSH as a cofactor to inhibit lipoxygenase-mediated lipid peroxidation [42,43]. Results of the present study showed that Ferrostatin-1 effectively increased the level of GSH in Balb/3T3 cells caused by CoNPs. In addition, Western blots showed that treatment of Balb/3T3 cells with CoNPs resulted in downregulation of GPX4 and SLC7A11 proteins, although Ferrostatin-1 could inhibit this decrease.

Cellular uptake of CoNPs is a prerequisite for development of toxicity. In the present study, TEM images revealed a marked deposition of CoNPs in the cytoplasm, but none in the nucleus. Moreover, it was evident that Ferrostatin-1 can prevent cobalt uptake by Balb/3T3 cells. Interestingly, Ferrostatin-1 could effectively suppress CoNPs-induced increase in iron (including Fe^2+^ and total iron concentration) levels in Balb/3T3 cells. This was consistent with findings from a previous study that concluded that ferritin degradation causes unstable iron overload and leads to ferroptosis [44]. Finally, we found that CoNPs treatment mediated a decrease in cell mitochondrial membrane potential and destruction of mitochondrial membrane structure in Balb/3T3 cells. Conversely, exposure to Ferrostatin-1 effectively reversed these phenomena, increasing mitochondrial membrane potential and maintaining the mitochondrial shape.

## Conclusion

Hip arthroplasty is usually accompanied by wearing out of the metal prosthesis and release of CoNPs. This release causes a series of local or systemic adverse reactions, which seriously affect the patient’s physical and mental health. Previous studies, exploring the detoxification mechanism of CoNPs, have only used some common antioxidants based on the oxidative stress mechanism. In the present study, we demonstrated that CoNPs can cause ferroptosis in Balb/3T3 cells, while ferroptosis inhibitor, Ferrostatin-1, can effectively protect the cells from this toxicity. Taken together, our findings lay a foundation for further exploration into the signaling pathways that regulate CoNP-induced ferroptosis in Balb/3T3 cells, and are also expected to guide *in vivo* experiments.
